# Suicide Attempt by a Corrosive Agent Causing Unusual Outcomes and Complications

**DOI:** 10.7759/cureus.42861

**Published:** 2023-08-02

**Authors:** Yuli Breier, Robert Kushmakov, Wesley D Banks, Bo Guan, Danielle Langan

**Affiliations:** 1 Emergency Department, Touro College of Osteopathic Medicine, New York, USA; 2 Emergency Department, Staten Island University Hospital, Northwell Health, Staten Island, USA

**Keywords:** corrosive poisoning, medical toxicology, suicide attempt, critical emergency medicine, caustic ingestion injury

## Abstract

We commonly encounter patients in the emergency department who present after a suicide attempt. The methods can vary and present unique challenges depending on the nature of the attempt. We present an unsuccessful attempt via chemical ingestion that led to severe complications involving the ingestion of drain cleaner with both highly corrosive and caustic properties. The management and presentation are discussed in great detail to further investigate the best treatment plan for both acute and chronic complications.

## Introduction

In the United States, about 45,000 people die by suicide each year [1]. By far, the most common method of suicide in the United States is by using firearms and most prominently by males [2]. There is not enough data about corrosive substances being used as a successful method of suicide; however, the damage and disability they cause are severe and underreported. Ingestion of a caustic agent can lead to the development of various gastrointestinal pathologies such as strictures, perforation, bleeding, and to a greater extent multiorgan system failure, disseminated intravascular coagulation, and sepsis. Rapid interventions in the emergency department (ED) during the acute phase of ingestion can improve outcomes and save lives. Nevertheless, the outcomes of ingesting caustic agents remain devastating, with many patients requiring an esophagostomy and lengthy recovery [3].

## Case presentation

A 68-year-old man with an unknown past medical history was brought to the ED via ambulance, accompanied by his granddaughter. At the time of arrival, the patient could not provide any information due to severe respiratory distress and edema of his oropharynx. According to his granddaughter, the patient had swallowed a sodium hydroxide-containing drain cleaner in a suicide attempt. On presentation, the patient had a muffled voice and a swollen tongue and was not able to tolerate his excessive secretions. Otolaryngology was consulted for visualization of his upper airway which revealed a significant caustic burn injury to the entire larynx (base of the tongue, vallecula, epiglottis, false vocal cords, arytenoids, and hypopharynx) with pooling of secretions within the larynx. Rapid-sequence intubation was subsequently performed. After stabilization in the ED, the patient was admitted to the intensive care unit for further treatment and monitoring.

**Figure 1:**
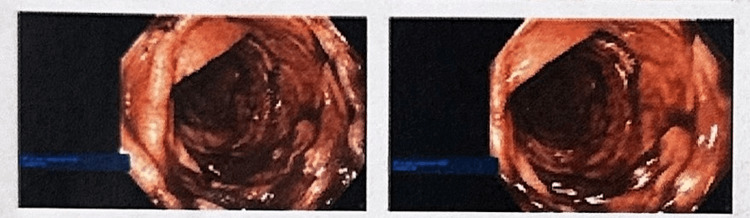
Severely damaged scattered areas of necrosis and ulcerations can be seen starting from 25 cm throughout the esophagus (Grade 3A/3B).

Over the following two days post-admission, the patient underwent an endoscopy and bronchoscopy which revealed damage to the left main stem bronchus. Operative findings included liquefactive necrosis (grade 3A/3B) throughout the esophagus (Figure 1) and the dependent portion of the stomach with no evidence of perforation. A mucosal injury was observed in the duodenal bulb; however, the extent of the injury was less pronounced compared to the esophagus. Normal mucosa was noted in the second part of the duodenum. On the fourth day of hospitalization, the patient required an emergent diagnostic laparoscopy, during which two small areas of ischemia without necrosis were identified. The patient underwent right video-assisted thoracic surgery with distal esophagectomy, creation of cervical esophagostomy, laparoscopic subtotal gastrectomy, gastrostomy and jejunostomy tube (J tube) placement, and tracheostomy. The patient tolerated the procedure well and was transferred to the cardiothoracic unit (CTU) for further management. In the CTU, the patient remained on the ventilator through his tracheostomy. He was eventually started on feeds through his J tube which he tolerated well. Throughout his time in the CTU, he was weaned off sedation and the ventilator and was able to ventilate through his tracheostomy independently. On the 17th day of hospitalization, another bronchoscopy and esophagoscopy were performed to visualize the healing process, which was noted to be improving. The patient’s hospital course was complicated by new-onset atrial fibrillation with rapid ventricular response, which was eventually controlled with oral medications. Additionally, feeds were gradually increased over time and the tracheostomy tube was eventually decannulated. Our patient was able to communicate, use self-suction, ambulate, and breathe regularly on room air before discharge. After a complete 40-day hospitalization, our patient was discharged and received coordinated care at an assisted nursing facility.

## Discussion

Ingestion of a corrosive agent may present with clinical symptoms such as nausea, vomiting, hoarseness, and drooling. Visible lesions such as superficial ulcers of the lip, buccal mucosa, tongue, and palate are prominent as well [4]. However, around 30% of patients who ingest a caustic agent do not present with classical symptoms [5]. This is concerning because patients may be evaluated and discharged before symptoms begin to manifest. Immediately after a patient presents with ingestion of a caustic agent, the airway should be evaluated. If there are concerning signs and symptoms present on examination, the airway should be protected via intubation. Immediately after, the patient should be evaluated for esophageal perforation. If vomiting, drooling, or stridor is present, or perforation is suspected, immediate endoscopy is needed for evaluation [6,7]. If no suspicion of perforation is initially present, computerized tomography (CT) imaging should be ordered to rule out perforation. If there is a perforation, surgery is indicated. Overall, if the patient has no perforation, management consists of nothing by mouth, trial via feeding tube, and follow-up esophagogastroduodenoscopy (EGD) within one to two weeks [7].

A retrospective analysis was done in 2017 evaluating patients after caustic agent ingestion and the role of early endoscopic evaluation in decreasing morbidity, mortality, and cost. In this study, 6,011 patients were evaluated, where 43% underwent early upper gastrointestinal endoscopy (EaEN) in 24 hours, 40% underwent EaEN in 48 hours, and 17% underwent late gastrointestinal endoscopy (LaEN). The LaEN group was found to have an associated three-fold increase in poor clinical outcomes compared to the EaEN groups. An even bigger increase (four-fold) was found for cost admission, and a five-fold increase in prolonged admissions when compared to the EaEN group. Furthermore, the EaEN group that underwent EaEN in 24 hours versus 48 hours had no significant difference in clinical outcomes [8]. Approaching the 72-hour mark, the corrosive lesions enter a stage known as the ulceration granulation phase. This is where necrotic tissue can begin to slough and leave an ulcerative base. This can increase the risk of perforation when performing EGD [9]. Therefore, it is often recommended to perform EGD within 24-48 hours of ingestion of a caustic agent.

It is important for clinicians to be aware of differences between alkali and acidic ingestions in terms of severity and injury pattern. Some common caustic ingestions are outlined in Table 1 [10]. Alkali ingestion tends to cause liquefactive necrosis, while acidic ingestion tends to cause coagulative necrosis [11]. Akali ingestions typically result in greater injury to the patient. Alkali fluids bond with tissue protein rapidly on contact often resulting in corrosiveness in the esophagus. Acidic substances, alternatively, tend to damage the stomach lining. Because acidic substances have less surface tension, they often bypass the esophagus, with a less likely or less severe chance of damaging the esophagus [12]. Long-term sequelae of alkali ingestion include strictures of the esophagus, odynophagia, dysphagia, malnutrition, and even cancer. Cancer can arise decades after the original ingestion or squamous cell carcinoma complication of Grade 3 esophageal caustic ingestion [11,13,14].

**Table 1:** Alkali versus acidic agents.

	Caustic ingestions
Acid	Toilet bowl cleaners, swimming pool agents, rust removers (sulfuric acid, hydrochloric acid, nitric acid)
Alkali	Oven cleaners, liquid agents, liquid drain cleaners (sodium hydroxide, potassium hydroxide), hair products (calcium and lithium hydroxide), household cleaners (ammonium)

## Conclusions

Accidental and non-accidental caustic substance ingestion should never be taken lightly. When discussing caustic ingestions, evaluation of what agent was used and understanding the symptoms and their progression are essential to treat patients effectively. Providers must be mindful of the time of ingestion, time of presentation to the ED, and when immediate intervention is indicated for ingestion of a caustic agent.

## References

[1] (2018). Centers for Disease Control and Prevention. Fatal injury and violence data. Reports.

[2] Bachmann S (2018). Epidemiology of suicide and the psychiatric perspective. Int J Environ Res Public Health.

[3] Hall AH, Jacquemin D, Henny D, Mathieu L, Josset P, Meyer B (2019). Corrosive substances ingestion: a review. Crit Rev Toxicol.

[4] Levsky ME, Miller MA (2007). Isopropyl alcohol skin prep pads: the extreme case. J Emerg Med.

[5] Methasate A, Lohsiriwat V (2018). Role of endoscopy in caustic injury of the esophagus. World J Gastrointest Endosc.

[6] Hoffman RS, Burns MM, Gosselin S (2020). Ingestion of caustic substances. N Engl J Med.

[7] Chen YJ, Seak CJ, Cheng HT (2022). Evaluation of a diagnostic and management algorithm for adult caustic ingestion: new concept of severity stratification and patient categorization. J Pers Med.

[8] Abbas A, Brar TS, Zori A, Estores DS (2017). Role of early endoscopic evaluation in decreasing morbidity, mortality, and cost after caustic ingestion: a retrospective nationwide database analysis. Dis Esophagus.

[9] Dafoe CS, Ross CA (1969). Acute corrosive oesophagitis. Thorax.

[10] Lupa M, Magne J, Guarisco JL, Amedee R (2009). Update on the diagnosis and treatment of caustic ingestion. Ochsner J.

[11] Judkins DG, McTeer AV (2022). Alkali Toxicity. McTeer, Alkali Toxicity, in StatPearls.

[12] Park KS (2014). Evaluation and management of caustic injuries from ingestion of acid or alkaline substances. Clin Endosc.

[13] Srivatsav A, Ghanayem R, Dahdal S, Khalaf N (2020). Treatment of esophageal stricture after lye ingestion. ACG Case Rep J.

[14] Zhang X, Wang M, Han H, Xu Y, Shi Z, Ma G (2012). Corrosive induced carcinoma of esophagus after 58 years. Ann Thorac Surg.

